# Postextubation dysphagia is persistent and associated with poor outcomes in survivors of critical illness

**DOI:** 10.1186/cc10472

**Published:** 2011-09-29

**Authors:** Madison Macht, Tim Wimbish, Brendan J Clark, Alexander B Benson, Ellen L Burnham, André Williams, Marc Moss

**Affiliations:** 1 Division of Pulmonary Sciences and Critical Care Medicine, University of Colorado Denver, 12700 East 19th Avenue, Aurora, CO 80045, USA; 2 Rehabilitation Therapy, University of Colorado Hospital, 12700 East 19th Avenue, Aurora, CO 80045, USA; 3 Division of Biostatistics and Bioinformatics, National Jewish Health, 1400 Jackson Street, Denver, CO 80206, USA

## Abstract

**Introduction:**

Dysphagia is common among survivors of critical illness who required mechanical ventilation during treatment. The risk factors associated with the development of postextubation dysphagia, and the effects of dysphagia on patient outcomes, have been relatively unexplored.

**Methods:**

We conducted a retrospective, observational cohort study from 2008 to 2010 of all patients over 17 years of age admitted to a university hospital ICU who required mechanical ventilation and subsequently received a bedside swallow evaluation (BSE) by a speech pathologist.

**Results:**

A BSE was performed after mechanical ventilation in 25% (630 of 2,484) of all patients. After we excluded patients with stroke and/or neuromuscular disease, our study sample size was 446 patients. We found that dysphagia was present in 84% of patients (*n *= 374) and classified dysphagia as absent, mild, moderate or severe in 16% (*n *= 72), 44% (*n *= 195), 23% (*n *= 103) and 17% (*n *= 76), respectively. In univariate analyses, we found that statistically significant risk factors for severe dysphagia included long duration of mechanical ventilation and reintubation. In multivariate analysis, after adjusting for age, gender and severity of illness, we found that mechanical ventilation for more than seven days remained independently associated with moderate or severe dysphagia (adjusted odds ratio (AOR) = 2.84 [interquartile range (IQR) = 1.78 to 4.56]; *P *< 0.01). The presence of severe postextubation dysphagia was significantly associated with poor patient outcomes, including pneumonia, reintubation, in-hospital mortality, hospital length of stay, discharge status and surgical placement of feeding tubes. In multivariate analysis, we found that the presence of moderate or severe dysphagia was independently associated with the composite outcome of pneumonia, reintubation and death (AOR = 3.31 [IQR = 1.89 to 5.90]; *P *< 0.01).

**Conclusions:**

In a large cohort of critically ill patients, long duration of mechanical ventilation was independently associated with postextubation dysphagia, and the development of postextubation dysphagia was independently associated with poor patient outcomes.

## Introduction

Acute respiratory failure (ARF) is a heterogeneous disorder that frequently requires admission to an ICU and the initiation of mechanical ventilation. The annual incidence of US patients who require mechanical ventilation is approximately 300,000 [1]. On the basis of an ARF case fatality rate of 27%, there are an estimated 220,000 survivors of mechanical ventilation each year [2]. These patients have a median duration of survival of more than 5 years and suffer from pulmonary dysfunction, cognitive impairment and decreased quality of life [3-6].

Recently, increasing attention has been focused on the debilitating effects of neuromuscular dysfunction among ARF survivors [7,8]. Although peripheral muscle weakness is one form of neuromuscular dysfunction that has been independently associated with mortality [9], an underrecognized form is swallowing dysfunction. Also known as "dysphagia," swallowing dysfunction is the inability to effectively transfer food and liquid from the mouth into the stomach. The consequences of dysphagia in non-critically ill, neurologically impaired patients include aspiration, pneumonia, malnutrition, placement of feeding tubes, decreased quality of life, increased institutional care and increased mortality [10-12].

The development of dysphagia has been reported to be common among ARF survivors, with estimates ranging from 3% to 62% in a recent meta-analysis [13]. Although known risk factors for dysphagia in non-critically ill patients include stroke and neuromuscular dysfunction, the risk factors for the development of postextubation dysphagia have been relatively unexplored [12,14-16]. The duration of mechanical ventilation was associated with dysphagia in two studies [17,18]; however, other work has shown these characteristics to be unrelated [19-21]. In addition, the effects of swallowing dysfunction on hospital outcomes such as length of stay, pneumonia and reintubation are also relatively unknown. Therefore, we sought to identify specific risk factors associated with dysphagia in these patients and to define the effects of postextubation dysphagia on outcomes in ARF patients.

## Materials and methods

### Study design

Using the University of Colorado Hospital medical records system, we conducted a retrospective, observational cohort study of ICU survivors who had undergone a bedside swallow evaluation (BSE) by a speech pathologist. Patients were eligible if they met all of the following criteria: (1) admission to any ICU during the two-year period from April 2008 to April 2010, (2) mechanical ventilation for any duration, (3) BSE by a speech pathologist and (4) older than 17 years of age. We included patients who received short-duration mechanical ventilation (less than 48 hours), as previous authors have suggested that even short-term endotracheal intubation may cause swallowing dysfunction [22,23]. The decision to consult a speech pathologist was left to the discretion of the primary treating physicians. Patients were excluded if they (1) had an acute or preexisting diagnosis of either a neuromuscular disease or a cerebrovascular accident (CVA) or (2) received their first BSE prior to the initiation of mechanical ventilation. The Colorado Multiple Institutional Review Board approved both the study protocol and a waiver of informed consent.

### Data collection

Patients who had been assessed using a BSE were identified in a speech-language pathology database. Data were abstracted from various components of the medical records, including admission and progress notes, discharge summaries, ICU flow sheets, laboratory and radiological data and internal diagnostic coding.

### Data analysis

Our first analysis was to determine the risk factors for the presence of swallowing dysfunction. In this analysis, the primary independent variable of interest was the duration of mechanical ventilation, and secondary variables of interest included reintubation, endotracheal tube size and severity of illness. Duration of mechanical ventilation was calculated using the hospital database. Endotracheal tube size was recorded from a respiratory therapy database and corresponded to the internal diameter of the endotracheal tube in millimeters. Severity of illness was measured using the Sequential Organ Failure Assessment (SOFA) score and was calculated at the time of admission to the ICU. The partial pressure of arterial oxygen to fraction of inspired oxygen (PaO_2_/FiO_2_) ratio was corrected for the altitude and mean atmospheric pressure in Denver (PaO_2_/FiO_2 _SOFA score = (PaO_2_/FiO_2 _Denver) ÷ 0.826). We omitted the component of the SOFA score corresponding to the Glasgow Coma Scale (GCS) score, as these data were not routinely available. When examining reintubation, we recorded the timing of reintubation in relation to the initial BSE.

Our primary outcome variable for this analysis was the presence of swallowing dysfunction as determined by certified speech pathologists. BSEs consisted of (1) patient history; (2) examination of oral, laryngeal and vocal cord swallowing exercises; (3) swallowing trials with different food and liquid consistencies; and (4) assessment of swallowing function with various compensatory techniques. Speech pathologists used the Dysphagia Outcome and Severity Scale (DOSS), which has been reported to correlate with findings on videofluoroscopic studies of swallowing (VFSS) [24]. "Normal swallowing" was defined as the absence of supraglottic penetration or aspiration (DOSS score = 7), "mild dysphagia" was defined as intermittent evidence of a trace of supraglottic penetration (DOSS score = 5 or 6), "moderate dysphagia" was defined as two or fewer instances of supraglottic penetration with a single food or liquid consistency (DOSS score 3 or 4) and "severe dysphagia" was defined as frank aspiration of more than one food or liquid consistency (DOSS score 1 or 2). The food and liquid consistencies used by the speech pathologists were consistent with published diets described by the American Dietetics Association National Dysphagia Diet Task Force [25]. The decision to perform a VFSS was made by either the speech pathologist or the treating physician. When performed, VFSS were interpreted primarily by radiologists and dysphagia severity was judged by treating speech pathologists, on the basis of the eight-point Penetration-Aspiration Scale [26]. For patients who were assessed on the basis of both a BSE and a VFSS, the VFSS score was used to determine dysphagia severity. In our study, a Penetration-Aspiration Scale score of 1 indicated normal swallowing, 2 or 3 indicated mild dysphagia, 4 or 5 indicated moderate dysphagia and 6 to 8 indicated severe dysphagia. Given some interobserver variability for moderate and severe dysphagia in the initial study validating the DOSS [24] and the lack of validation of these scores in ICU patients, we combined moderate and severe dysphagia into one category for subsequent analyses.

In our second analysis, we attempted to determine the effect of the presence of swallowing dysfunction on a variety of outcome variables, including the need for reintubation, the development of hospital-acquired pneumonia (HAP), hospital length of stay, surgical placement of a feeding tube and in-hospital mortality. For this analysis, our primary independent variable of interest was the presence of swallowing dysfunction as defined above. Our outcome variables were defined using the following criteria. "Reintubation" was defined as the placement of an endotracheal tube for any reason after the initial endotracheal tube was removed. The diagnosis of HAP required the presence of criteria defined in the American Thoracic Society/Infectious Diseases Society of America guidelines [27] as well as the decision of the treating physician to administer antimicrobial treatment. For hospital length of stay, we recorded both total hospital days and the time spent in the hospital after the initial BSE. "Feeding tube placement" was defined as the surgical placement of a gastric or jejunal tube by a surgeon, gastroenterologist or interventional radiologist. We predetermined that the most clinically relevant outcomes were (1) the development of pneumonia, (2) the need for reintubation and (3) in-hospital mortality. In addition to analyzing each variable separately, we created a composite outcome of these three variables. We recorded the existence of the composite outcome if any one of these three variables was present. For the purposes of our analyses, patients with the presence of only one outcome were treated the same as patients with the presence of two or three outcomes.

### Statistical analysis

Data that were not normally distributed are reported as medians [25th to 75th interquartile ranges]. Univariate comparisons were evaluated using the χ^2 ^test or Kruskal-Wallis test as appropriate. Nonparametric tests were used when data were not normally distributed. Backward logistic regression models were used to determine the effect of the duration of mechanical ventilation on the presence of dysphagia and the effect of dysphagia on patient outcomes. Because of the known strong effects of tracheostomy on swallowing function and a potential interaction between tracheostomy and duration of mechanical ventilation in the models we used to examine the effect of the duration of mechanical ventilation on the presence of dysphagia, we prespecified that we would perform separate multivariate analyses for patients with or without tracheostomy. SAS version 9.1 software (SAS Institute Inc, Cary, NC, USA) was used for all analyses, and *P *< 0.05 was considered statistically significant. Confidence intervals (95%) for adjusted odds ratios (AORs) and 25th to 75th interquartile ranges [IQRs] for median values are recorded in square brackets. The Bonferroni correction for multiple comparisons was performed were appropriate.

## Results

**Figure 1:**
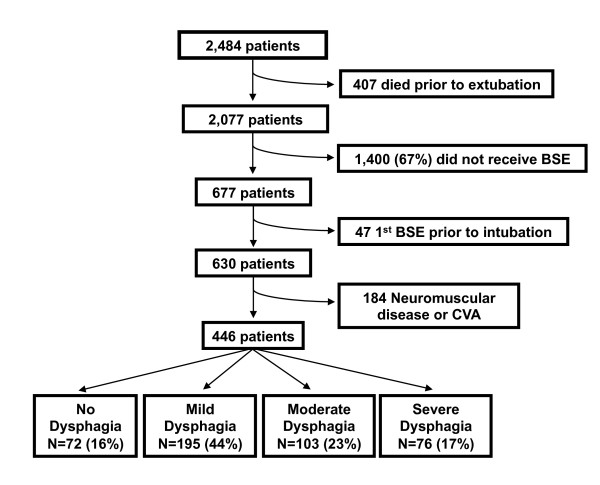
**Flowchart detailing enrollment of subjects**. BSE = bedside swallow evaluation; CVA = cerebrovascular accident.

The study enrollment process is outlined in Figure 1. Of the 2,484 patients who met the inclusion criteria, 407 died prior to extubation. Of the remaining patients, 67% (1,400 of 2,077) had not been assessed by BSE. A physician's order to perform a BSE was most common for patients on a neurological service (45%), followed a medical service (34%) and a surgical service (17%) (*P *< 0.001). Compared to patients who were not assessed by BSE, patients who were assessed by BSE were more likely to have had a tracheostomy (44% vs 25%; *P *< 0.001), a longer duration of mechanical ventilation (7 days [3 to 14] vs 2 days [1 to 5]; *P *< 0.001) and a longer ICU stay (10 days [5 to 19] vs 3 days [2 to 7]; *P *< 0.001). Of the remaining 677 patients who were assessed by BSE during their hospital stay, 47 were excluded because the initial BSE had been done prior to intubation and 184 were excluded because they had a diagnosis of CVA or neuromuscular disease. The remaining 446 patients were included in our final analysis. Exactly half of these patients were cared for in a medical ICU, 34% received care in a surgical ICU, 9% were treated in a neurologic ICU and 7% were cared for in a cardiac ICU. Approximately two-thirds of these patients had an underlying medical disease. Some degree of dysphagia was present in 84% (374 of 446) of those patients selected to be assessed by BSE, in 18% (374 of 2,077) of the total population of ARF survivors and in 15% (374 of 2,484) of all patients admitted to the ICU during the study period. Among the 446 patients included in the study, dysphagia severity was mild in 44% (*n *= 195), moderate in 23% (*n *= 103) and severe in 17% (*n *= 76). In-hospital mortality in this selected cohort of ARF survivors was 7.6%. Only 11 (2.5%) of the 446 study patients received a modified barium swallow in addition to a BSE.

Univariate analyses performed to evaluate patient characteristics associated with the presence of postextubation dysphagia are described in Table 1. Statistically significant risk factors for severe dysphagia included long duration of mechanical ventilation, reintubation, tracheostomy and male gender. In multivariate analysis, owing to an interaction between tracheostomy and duration of mechanical ventilation, we performed two separate analyses for those patients with versus without tracheostomy. In the analysis of patients without tracheostomy, after adjusting for age, gender and severity of illness, mechanical ventilation for more than seven days remained independently associated with moderate or severe dysphagia (AOR 2.84 [1.78 to 4.56]; *P *< 0.01). In the analysis of patients with tracheostomy, mechanical ventilation for more than seven days was not independently associated with moderate or severe dysphagia.

**Table 1:** Univariate analysis of risk factors for postextubation dysphagia

	Dysphagia severity			
Risk factors	None (*n *= 72)	Mild (*n *= 195)	Moderate or severe (*n *= 179)	*P *value
Age (years)	50 ± 15	52 ± 17	54 ± 17	0.22
Male sex	52 (72)	108 (55)	109 (61)	0.04
Weight (kg)	80 ± 20	83 ± 24	78 ± 23	0.10
Comorbidities, *n *(%)				
Acute MI	8 (11)	20 (10)	23 (13)	0.73
Heart failure	15 (21)	44 (23)	28 (16)	0.22
COPD	14 (19)	34 (17)	36 (20)	0.80
Diabetes mellitus	19 (26)	54 (28)	41 (23)	0.56
OSA	24 (33)	63 (32)	65 (36)	0.71
Pneumonia (before BSE)	32 (44)	69 (35)	79 (44)	0.17
SOFA score (without GCS score)	3.5 [2 to 6]	4 [2 to 5]	3 [2 to 5]	0.98
Tracheostomy^a^	3 (4)	25 (13)	50 (28)	<0.01
Endotracheal tube size, *n *(%)				0.17
7.0 mm or less (*n *= 74)	10 (14)	39 (20)	25 (15)	
7.5 mm (*n *= 108)	23 (32)	38 (20)	47 (28)	
8.0 mm or greater (*n *= 251)	39 (54)	114 (60)	98 (58)	
Intubated in ED, *n *(%)	15 (21)	28 (14)	32 (18)	0.41
Reintubation (before BSE), *n *(%)^a^	10 (14)	17 (9)	43 (24)	<0.01
Ventilator days	4 [2 to 7]	6 [3 to 11]	10 [5 to 17]	<0.01
Mechanical ventilation more than 7 days, *n *(%)^a,b^	15 (21)	77 (39)	105 (59)	<0.01

BSE = bedside swallow evaluation; COPD = chronic obstructive pulmonary disease; ED = emergency department; GCS = Glasgow Coma Scale; MI = myocardial infarction; OSA = obstructive sleep apnea; SD = standard deviation; SOFA = Sequential Organ Failure Assessment. Data are presented as *n *(%), means ± SD or medians [25th to 75th percentiles]. ^a^*P *< 0.05 for comparison of moderate or severe dysphagia to no dysphagia after Bonferroni correction. ^b^*P *< 0.05 for comparison of mild to no dysphagia after Bonferroni correction.

Among the 243 patients whose dysphagia resolved while they were in the hospital, the median duration of dysphagia was 3 days [2 to 6 days] for those with mild dysphagia (*n *= 162) and 6 days [4 to 12 days] for those with moderate or severe dysphagia (*n *= 81). At the time of hospital discharge, dysphagia was present in more patients with moderate or severe dysphagia compared to those with mild dysphagia (55% (98 of 179) vs 17% (33 of 195); *P *< 0.0001).

**Figure 2:**
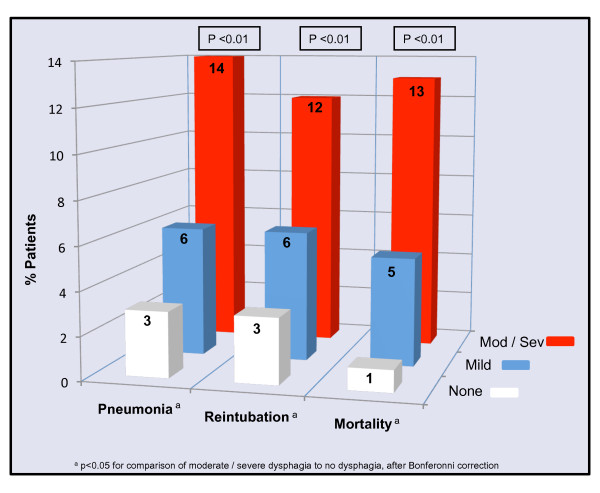
**Association between dysphagia severity and pneumonia, reintubation and mortality**.

Univariate analyses performed to evaluate associations between the presence and severity of dysphagia and hospital outcomes are shown in Table 2 and Figure 2. The presence of dysphagia was significantly associated with the number of hospital days after the initial BSE, discharge status, no oral intake (NPO) status, surgical placement of a feeding tube and composite outcome of pneumonia, reintubation or in-hospital mortality (Table 2). Dysphagia was also independently and significantly associated with pneumonia, reintubation and in-hospital mortality (Figure 2). In multivariate analysis, after adjusting for age and severity of illness, the presence of moderate or severe dysphagia was independently associated with the composite outcome of pneumonia, reintubation or death (AOR 3.31 [1.89 to 5.90]; *P *< 0.01).

**Table 2:** Univariate analysis of patient outcomes by severity of dysphagia

Outcomes	None (*n *= 72)	Mild (*n *= 195)	Moderate or severe (*n *= 179)	*P *value
Hospital days after BSE^a^	5 [3 to 8]	7 [5 to 12]	8 [5 to 15]	<0.01
Discharge to home^a,b^	54 (75)	100 (51)	69 (39)	<0.01
Dysphagia at discharge^a-c^	0 (0)	33 (17)	98 (55)	<0.01
Kept NPO^a-c^	0 (0)	29 (15)	132 (74)	<0.01
Surgical feeding tube^a,c^	0 (0)	10 (5)	26 (15)	<0.01
Pneumonia, reintubation and death^a,c,d^	4 (6)	22 (11)	49 (27)	<0.01

BSE = bedside swallow evaluation; NPO = no oral intake. Data are presented as *n *(%) or medians [25th to 75th percentiles]. ^a^*P *< 0.05 for comparison of moderate or severe dysphagia to no dysphagia after Bonferroni correction. ^b^*P *< 0.05 for comparison of mild to no dysphagia after Bonferroni correction. ^c^*P *< 0.05 for comparison of moderate or severe dysphagia to mild dysphagia after Bonferroni correction. ^d^Outcome data for pneumonia, reintubation and death are composite totals.

## Discussion

In a large group of critically ill patients, we have demonstrated that, among patients who were not assessed by BSE, both longer duration of mechanical ventilation and repeat intubation were associated with the development of dysphagia. In addition, we found that postextubation dysphagia often persists at the time of discharge and is associated with poor outcomes. Specifically, moderate or severe dysphagia is associated with an increased risk of reintubation, development of pneumonia, longer hospital stay, reduced dietary intake, placement of feeding tubes, discharge to a nursing home and increased risk of death.

The exact frequency of postextubation dysphagia among all medical and surgical ICU patients remains unknown. The primarily limitations on understanding this frequency are (1) the absence of a widely accepted diagnostic standard for dysphagia and (2) the relatively small populations represented in the existing studies. Barker and colleagues [17] conducted a retrospective chart review of 254 patients (including those with CVA) who required mechanical ventilation for more than 48 hours following cardiac surgery and found evidence of dysphagia in 130 patients (51%) on the basis of BSE. El Solh and colleagues [21] performed a fiberoptic endoscopic evaluation of swallowing (FEES) in 84 consecutive extubated medical ICU patients who did not have preexisting dysphagia, CVA or neuromuscular disease. In their study, aspiration occurred in 37 (44%) of 84 patients and was silent (that is, not associated with cough or patient discomfort and thus undetectable on BSE) in 11 of those 37 patients (13% of the total population). In a small study comparing FEES to BSE, Barquist *et al*. [19] randomized 70 recently extubated trauma surgery patients to receive either FEES or BSE and found evidence of aspiration in 5 (13.5%) of 37 in the FEES group and 2 (6%) of 33 in the BSE group. Ajemian and colleagues [28] performed FEES in 51 consecutive extubated surgical and medical ICU patients without a previous swallowing disorder and found aspiration in 27 (56%) of 48 patients, in 12 of whom it occurred silently (25% of the total). Importantly, over two-thirds of the patients in our initial cohort did not undergo an evaluation for dysphagia, thus the true incidence of dysphagia among the patients in our study is uncertain. However, postextubation dysphagia was present in 84% (374 of 446) of those patients selected to undergo a BSE, representing 18% (374 of 2,077) of the total population of ARF survivors, and 15% (374 of 2,484) of all ICU admissions. Further research, including prospective observational studies, is necessary to determine the true frequency of postextubation dysphagia.

The association between intubation duration and severity of dysphagia is supported by Barker *et al*.'s review [17] as well as by two studies in which patients intubated for cardiopulmonary bypass were examined [18,29]. However, this association has not been reported in other analyses [19,21,28,30]. Many factors could account for this discrepancy, namely, differences in sample size, event rate and intubation duration. While this association is plausible based on the likely increased degree of oral, pharyngeal and laryngeal damage in patients intubated for long periods, it also remains possible that short intubation duration is sufficient to cause dysphagia. The association between intubation duration and dysphagia, as well as the neuromotor and sensory mechanisms underlying swallowing dysfunction in newly extubated patients, needs to be further explored. Our study and that by Barker *et al*. [17] are the first to suggest that reintubation may be associated with the development of dysphagia, an association with potentially important applications for future dysphagia studies.

A recent review of the National Hospital Discharge Survey showed a significant association between dysphagia and both hospital length of stay and mortality [31], but few studies have examined the association between patient outcome and dysphagia severity among extubated medical and surgical ICU patients without CVA or neuromuscular disease. Both El Solh *et al*. [21] and Ajemian *et al*. [28] found neither postextubation aspiration pneumonia nor any deaths in their studies. In contrast, 14% of the patients with moderate or severe dysphagia in our cohort had aspiration pneumonia, and 13% died while in the hospital, suggesting that either our cohort had a higher severity of illness or the FEES-based diet modifications used in these studies prevented these complications. Barker *et al*. [17] showed an association between dysphagia and reintubation, longer hospital stay, NPO and the presence of feeding tubes, although they did not record data on mortality.

Our study has several limitations. Sixty-seven percent of patients who survived to be extubated in our study were not assessed by BSE. We could study only those patients who had been assessed by BSE. Second, inherent in the design of our single-center, retrospective, observational cohort study is an inability to draw conclusions about causation. Similarly, some very important variables were inconsistently charted or not charted at all, and thus were not available for our analysis. For example, we were unable to obtain (1) GCS data to include in the SOFA score, (2) a reliable marker of sedation at the time of swallow evaluation, (3) height data to calculate both body mass index and height/endotracheal tube diameter ratio [32] and (4) reliable data on alcohol and tobacco use. Additionally, investigators in one previous study of postextubation dysphagia were able to obtain information on preadmission functional status, such as activities of daily living and preadmission swallowing dysfunction [21]. Although we attempted to control for this with admission severity of illness as well as exclusion of all patients with a history of CVA or neuromuscular dysfunction, we were not able to reliably obtain this information and thus omitted it from our analysis. Similarly, because we did not have data on the presence of preexisting swallowing dysfunction, we were not able to exclude these patients from our analysis. This could have resulted in a falsely elevated number of patients classified as having postextubation dysphagia.

A second important limitation in this area of research is the lack of a firm diagnostic test to determine the presence or absence of dysphagia. Although the DOSS has been validated to correlate with dysphagia severity on the basis of VFSS [24], the score is ultimately based on the judgment of the treating speech pathologist. We acknowledge that this evaluation is inherently subjective. On the basis of studies in outpatients, FEES is likely a more sensitive measure of aspiration than either BSE or videofluoroscopy [33-35]. Relatively few patients in our cohort were assessed by VFSS, and no patients were assessed by FEES. Further studies are necessary to explore the diagnosis, causes and complications of postextubation dysphagia.

## Conclusions

We have demonstrated that in a large group of survivors of mechanical ventilation, the development of postextubation dysphagia is associated with poor outcomes, including pneumonia, reintubation and death. Additionally, long duration of mechanical ventilation and prior reintubation are associated with the development of postextubation dysphagia. Understanding the mechanisms that contribute to postextubation dysphagia and developing methods to further address this disorder might decrease morbidity among a significant percentage of these critically ill patients.

## Key messages

♦ Swallowing dysfunction that occurs after mechanical ventilation, also known as "postextubation dysphagia," is likely common in a large population of medical and surgical ICU patients without preexisting neuromuscular disease.

♦ The results of this study suggest an independent association between postextubation dysphagia and poor patient outcomes, including pneumonia, reintubation and death.

♦ This study is the largest, and one of the first, to show that long duration of mechanical ventilation is associated with the development of postextubation dysphagia.

♦ Postextubation dysphagia persists at the time of discharge in a large portion of patients (131 (29%) of 446 patients in our study).

♦ Postextubation dysphagia is an underrecognized and potentially costly form of impairment in survivors of critical illness. Further research into this disorder is needed to identify its epidemiology and pathophysiology as well as to develop diagnostic strategies and treatments.

## Abbreviations

AOR: adjusted odds ratio; ARF: acute respiratory failure; BMI: body mass index; BSE: bedside swallow evaluation; CVA: cerebrovascular accident; DOSS: Dysphagia Outcome and Severity Scale; ED: emergency department; FEES: flexible endoscopic evaluation of swallowing; GCS: Glasgow Coma Scale; SOFA: Sequential Organ Failure Assessment; VFSS: videofluoroscopic studies of swallowing.

## Competing interests

The authors declare that they have no competing interests.

## Authors' contributions

MM conceived the study and contributed to data analysis, data collection and manuscript preparation. MM conceived the study and contributed to the data analysis and manuscript preparation. TW participated in the study design and contributed to the data collection. BJC, ABB and ELB contributed to the study design, analysis and manuscript preparation. AW contributed to the statistical analysis and manuscript preparation. All authors read and approved the final manuscript.

## References

[B1] Behrendt CE Acute respiratory failure in the United States: incidence and 31-day survival Chest 2000 118 1100 1105 10.1378/chest.118.4.1100 11035684

[B2] Esteban A Anzueto A Frutos F Alía I Brochard L Stewart TE Benito S Epstein SK Apezteguía C Nightingale P Arroliga AC Tobin MJ for the Mechanical Ventilation International Study Group Characteristics and outcomes in adult patients receiving mechanical ventilation: a 28-day international study JAMA 2002 287 345 355 10.1001/jama.287.3.345 11790214

[B3] Garland A Dawson NV Altmann I Thomas CL Phillips RS Tsevat J Desbiens NA Bellamy PE Knaus WA Connors AF Jr for the SUPPORT Investigators Outcomes up to 5 years after severe, acute respiratory failure Chest 2004 126 1897 1904 10.1378/chest.126.6.1897 15596690

[B4] Myhren H Ekeberg Ø Stokland O Health-related quality of life and return to work after critical illness in general intensive care unit patients: a 1-year follow-up study Crit Care Med 2010 38 1554 1561 10.1097/CCM.0b013e3181e2c8b1 20473149

[B5] Orme JF Jr Romney JS Hopkins RO Pope D Chan KJ Thomsen G Crapo RO Weaver LK Pulmonary function and health-related quality of life in survivors of acute respiratory distress syndrome Am J Respir Crit Care Med 2003 167 690 694 10.1164/rccm.200206-542OC 12493646

[B6] Hopkins RO Weaver LK Collingridge D Parkinson RB Chan KJ Orme JF Jr Two-year cognitive, emotional, and quality-of-life outcomes in acute respiratory distress syndrome Am J Respir Crit Care Med 2005 171 340 347 10.1164/rccm.200406-763OC 15542793

[B7] De Jonghe B Sharshar T Lefaucheur JP Authier FJ Durand-Zaleski I Boussarsar M Cerf C Renaud E Mesrati F Carlet J Raphaël JC Outin H Bastuji-Garin S Groupe de Réflexion et d'Etude des Neuromyopathies en Réanimation Paresis acquired in the intensive care unit: a prospective multicenter study JAMA 2002 288 2859 2867 10.1001/jama.288.22.2859 12472328

[B8] Herridge MS Tansey CM Matté A Tomlinson G Diaz-Granados N Cooper A Guest CB Mazer CD Mehta S Stewart TE Kudlow P Cook D Slutsky AS Cheung AM Canadian Critical Care Trials Group Functional disability 5 years after acute respiratory distress syndrome N Engl J Med 2011 364 1293 1304 10.1056/NEJMoa1011802 21470008

[B9] Ali NA O'Brien JM Jr Hoffmann SP Phillips G Garland A Finley JC Almoosa K Hejal R Wolf KM Lemeshow S Connors AF Jr Marsh CB Midwest Critical Care Consortium Acquired weakness, handgrip strength, and mortality in critically ill patients Am J Respir Crit Care Med 2008 178 261 268 10.1164/rccm.200712-1829OC 18511703

[B10] Ekberg O Hamdy S Woisard V Wuttge-Hannig A Ortega P Social and psychological burden of dysphagia: its impact on diagnosis and treatment Dysphagia 2002 17 139 146 10.1007/s00455-001-0113-5 11956839

[B11] Marik PE Aspiration pneumonitis and aspiration pneumonia N Engl J Med 2001 344 665 671 10.1056/NEJM200103013440908 11228282

[B12] Martino R Foley N Bhogal S Diamant N Speechley M Teasell R Dysphagia after stroke: incidence, diagnosis, and pulmonary complications Stroke 2005 36 2756 2763 10.1161/01.STR.0000190056.76543.eb 16269630

[B13] Skoretz SA Flowers HL Martino R The incidence of dysphagia following endotracheal intubation: a systematic review Chest 2010 137 665 673 10.1378/chest.09-1823 20202948

[B14] Herrera LJ Correa AM Vaporciyan AA Hofstetter WL Rice DC Swisher SG Walsh GL Roth JA Mehran RJ Increased risk of aspiration and pulmonary complications after lung resection in head and neck cancer patients Ann Thorac Surg 2006 82 1982 1988 10.1016/j.athoracsur.2006.06.018 17126095

[B15] Smith-Hammond CA New KC Pietrobon R Curtis DJ Scharver CH Turner DA Prospective analysis of incidence and risk factors of dysphagia in spine surgery patients: comparison of anterior cervical, posterior cervical, and lumbar procedures Spine (Phila Pa 1976) 2004 29 1441 1446 10.1097/01.BRS.0000129100.59913.EA 15223936

[B16] Ward EC Bishop B Frisby J Stevens M Swallowing outcomes following laryngectomy and pharyngolaryngectomy Arch Otolaryngol Head Neck Surg 2002 128 181 186 11843728 10.1001/archotol.128.2.181

[B17] Barker J Martino R Reichardt B Hickey EJ Ralph-Edwards A Incidence and impact of dysphagia in patients receiving prolonged endotracheal intubation after cardiac surgery Can J Surg 2009 52 119 124 19399206 PMC2663495

[B18] Rousou JA Tighe DA Garb JL Krasner H Engelman RM Flack JE Deaton DW Risk of dysphagia after transesophageal echocardiography during cardiac operations Ann Thorac Surg 2000 69 486 490 10.1016/S0003-4975(99)01086-3 10735685

[B19] Barquist E Brown M Cohn S Lundy D Jackowski J Postextubation fiberoptic endoscopic evaluation of swallowing after prolonged endotracheal intubation: a randomized, prospective trial Crit Care Med 2001 29 1710 1713 10.1097/00003246-200109000-00009 11546969

[B20] de Larminat V Montravers P Dureuil B Desmonts JM Alteration in swallowing reflex after extubation in intensive care unit patients Crit Care Med 1995 23 486 490 10.1097/00003246-199503000-00012 7874899

[B21] El Solh A Okada M Bhat A Pietrantoni C Swallowing disorders post orotracheal intubation in the elderly Intensive Care Med 2003 29 1451 1455 10.1007/s00134-003-1870-4 12904855

[B22] Heffner JE Swallowing complications after endotracheal extubation: moving from "whether" to "how." Chest 2010 137 509 510 10.1378/chest.09-2477 20202944

[B23] Stauffer JL Olson DE Petty TL Complications and consequences of endotracheal intubation and tracheotomy: a prospective study of 150 critically ill adult patients Am J Med 1981 70 65 76 10.1016/0002-9343(81)90413-7 7457492

[B24] O'Neil KH Purdy M Falk J Gallo L The Dysphagia Outcome and Severity Scale Dysphagia 1999 14 139 145 10.1007/PL00009595 10341109

[B25] National Dysphagia Diet Task Force The National Dysphagia Diet: Standardization for Optimal Care 2002 Chicago: National Dysphagia Diet Task Force

[B26] Rosenbek JC Robbins JA Roecker EB Coyle JL Wood JL A penetration-aspiration scale Dysphagia 1996 11 93 98 10.1007/BF00417897 8721066

[B27] American Thoracic Society; Infectious Diseases Society of America Guidelines for the management of adults with hospital-acquired, ventilator-associated, and healthcare-associated pneumonia Am J Respir Crit Care Med 2005 171 388 416 15699079 10.1164/rccm.200405-644ST

[B28] Ajemian MS Nirmul GB Anderson MT Zirlen DM Kwasnik EM Routine fiberoptic endoscopic evaluation of swallowing following prolonged intubation: implications for management Arch Surg 2001 136 434 437 10.1001/archsurg.136.4.434 11296115

[B29] Hogue CW Jr Lappas GD Creswell LL Ferguson TB Sample M Pugh D Balfe D Cox JL Lappas DG Swallowing dysfunction after cardiac operations: associated adverse outcomes and risk factors including intraoperative transesophageal echocardiography J Thorac Cardiovasc Surg 1995 110 517 522 10.1016/S0022-5223(95)70249-0 7637370

[B30] Romero CM Marambio A Larrondo J Walker K Lira MT Tobar E Cornejo R Ruiz M Swallowing dysfunction in nonneurologic critically ill patients who require percutaneous dilatational tracheostomy Chest 2010 137 1278 1282 10.1378/chest.09-2792 20299629

[B31] Altman KW Yu GP Schaefer SD Consequence of dysphagia in the hospitalized patient: impact on prognosis and hospital resources Arch Otolaryngol Head Neck Surg 2010 136 784 789 10.1001/archoto.2010.129 20713754

[B32] François B Bellissant E Gissot V Desachy A Normand S Boulain T Brenet O Preux PM Vignon P Association des Réanimateurs du Centre-Ouest (ARCO) 12-h pretreatment with methylprednisolone versus placebo for prevention of postextubation laryngeal oedema: a randomised double-blind trial Lancet 2007 369 1083 1089 10.1016/S0140-6736(07)60526-1 17398307

[B33] Kelly AM Drinnan MJ Leslie P Assessing penetration and aspiration: how do videofluoroscopy and fiberoptic endoscopic evaluation of swallowing compare? Laryngoscope 2007 117 1723 1727 10.1097/MLG.0b013e318123ee6a 17906496

[B34] Kelly AM Leslie P Beale T Payten C Drinnan MJ Fibreoptic endoscopic evaluation of swallowing and videofluoroscopy: does examination type influence perception of pharyngeal residue severity? Clin Otolaryngol 2006 31 425 432 10.1111/j.1749-4486.2006.01292.x 17014453

[B35] Leder SB Espinosa JF Aspiration risk after acute stroke: comparison of clinical examination and fiberoptic endoscopic evaluation of swallowing Dysphagia 2002 17 214 218 10.1007/s00455-002-0054-7 12140648

